# Health Care Use and Barriers to Care for Chronic Inflammatory Diseases (CID) among First and Second Generation South Asian Immigrant Children and Parents in Ontario Canada

**DOI:** 10.3390/ijerph192114608

**Published:** 2022-11-07

**Authors:** Andrea Rishworth, Tiffany Cao, Ashika Niraula, Kathi Wilson

**Affiliations:** 1 Department of Geography, Geomatics and Environment, Faculty Geography, University of Toronto Mississauga, Mississauga, ON L5L 1C6, Canada; 2 CERC in Migration and Integration, Toronto Metropolitan University, Toronto, ON M5B 2K3, Canada

**Keywords:** immigrant health, chronic inflammatory disease (CIDS), health care access, barriers to care, Canada, Ontario

## Abstract

Although immigrants are disproportionately impacted by growing chronic inflammatory disease (CIDs) rates, yet suffer barriers to access health care, little attention has been given to their primary healthcare or specialist healthcare access as it relates to complex, chronic diseases in Canada, a country with universal health care. This study aims to investigate CID health care use and barriers to care among first- and second-generation immigrant South Asian children and parents in the Greater Toronto Area, Ontario. Drawing on analysis of 24 in depth interviews with children and parents (14 children, 10 parents), the results reveal that although CIDs disproportionately affects South Asian immigrants, they encounter health system, geographic, interpersonal, and knowledge barriers to access requisite care. These barriers exist despite participants having a GP, and are compounded further by limited familial systems, culturally insensitive care, and structural inequities that in some instances make parents choose between health access or other basic needs. Although all participants recognized the importance of specialized care, only 11 participants regularly accessed specialized care, creating new schisms in CID management. The findings suggest that a multisectoral approach that address individual and structural level socio-structural drivers of health inequities are needed to create more equitable healthcare access.

## 1. Introduction

Healthcare access and utilization disparities are known to exist among immigrant populations in the Global North [1,2,3,4]. In the Canadian context, studies consistently demonstrate immigrants experience barriers to access health care related to long wait times, inconvenient hours of service provision, and financial concerns [5]. At the same time, immigrants’ experience barriers unique to their immigrant identity such as linguistic barriers, health system discrimination along with cultural variations that compound access challenges and experiences [6,7,8,9,10]. This means that immigrants seeking care often face complex challenges that may not be adequately addressed by targeting barriers pertaining to the general population [1,11].

This is particularly important in the context of chronic inflammatory diseases (CIDs) in Canada where health care utilization has been sparsely examined, despite first and second-generation immigrants being at elevated risk of developing a CID and requiring short- and long-term care [12]. Chronic inflammatory diseases (CIDs) are systemic disease of complex, multi-factorial etiology that that often lead that include inflammatory bowel disease (IBD), multiple sclerosis (MS), type 1 diabetes (T1D), systemic lupus erythematosus (SLE), rheumatoid arthritis (RA), ankylosing spondylitis (AS), and psoriasis and psoriatic arthritis (PPA) [13]. These diseases are known to inflict a variety of mental and physical health problems for individuals and are recognized among the top leading causes of death globally [14,15,16]. Thus, ongoing health care access and utilization is critical to effective CID management [13,14]. 

At a global scale, the incidence and prevalence of CIDs are rising on all continents, and within countries and populations where CIDs were essentially non-existent just several decades ago [12,13,17,18]. Within high income countries, the prevalence of CIDS is between 5–7%, increasingly most rapidly among immigrants from low to middle income countries [19,20]. Although CIDs were previously considered a set of diseases characteristic of Caucasians in Western industrialized countries, recent evidence suggests CIDs manifest in immigrants as early as first generation in countries with differential backgrounds to risk (i.e., low to high incidence countries and even high to low incidence countries) and becomes accentuated in subsequent generations [19,20,21,22,23]. While the pathological pathways of CIDs manifestations are unclear, the rapidity of change in their epidemiology suggests a strong contribution by non-genetic determinants and gene-environment interactions [12,13,17,18].

Within Canada, approximately five million people suffer from various CIDs [24]. While CIDs have been increasing among the general population, growing attention has centered on the development of CIDs among first- and second-generation immigrants in the country [25,26,27]. Studies demonstrate that although the prevalence of CIDs among first generation immigrants is lower than native born Canadians [18,25,28,29], immigrants who arrive to Canada at younger ages are at increased risk of CIDs; for every decade earlier in life that immigrants arrive in Canada, the risk of some CIDs increases by almost 10% [25]. Growing attention has also been given to CID variations within immigrant populations. One high risk, yet underappreciated group are immigrants of South Asian decent [12]. Compared to other immigrant groups in Canada, studies indicate that South Asian immigrant parents and their Canadian born children are at higher risk of developing a CID compared to other immigrant groups [12,13,18,25,30]. While reasons for these variations are unclear, altered risk of immune-mediated inflammation, particularly among South Asian Canadians suggests metagenomic multigenerational variations [18].

On top of differential epidemiology and risk factors are the uneven access to resources and health care services that shape the realities of CID progression and management. Although immigrants in Canada experience poorer health outcomes than non-immigrants [1,12], research consistently demonstrates that immigrants are more likely to have difficulties accessing health care services [1,31,32]. While the literature on access to care for chronic and complex diseases is sparce, though growing, recent studies highlight continued and signifigant disparities in access to care and health outcomes [33,34,35]. Hymen et al. [13] for instance examined health service use for type 2 diabetes among immigrants in Toronto and found that recent immigrants were significantly less likely than Canadian born groups to see a GP, a specialist, alternative care providers and less likely to report using dieticians, nurses and diabetes organizations as sources for disease information due to linguistic, financial, informational and systemic barriers to information and care. 

Harrington et al. [36], extend insight into these disparities, underscoring that both recent (0–9 years in Canada) and established immigrants (10+ years in Canada) are more likely to experience difficulties accessing general health care and specialist care than the Canadian population. While preventative care is critical to forestall disease development and manage disease progression, an important point from this study is that despite Canada’s Health Act that aims to ensure access to medically necessary health care services for all Canadians regardless of ability to pay, immigrants continue to face barriers accessing care services–all of which can contribute to feelings of dissatisfaction with the health care system and perpetuate poor self-care. Other studies consistently reveal immigrants have lower levels of disease awareness, disease risk factors and symptoms, often attributed to culturally inappropriate services, underscoring urgent gaps in chronic disease health services for immigrants [37,38,39]. These findings are worrying, particularly in the context of the ‘healthy’ immigrant effect whereby immigrants are healthier than their native born at the time of arrival in Canada; however, their health advantage often disappears within 10 years [40]. 

While the literature exploring immigrant children’s health care access is sparce, the studies that have been done suggest similar systemic barriers related to language, culture, finances, and relationships with health professionals [1,31,41,42]. Despite this growing attention, there are “still gaps in our knowledge of the health of immigrant children in Canada and this population still experiences challenges with healthcare access and disparate health outcomes across various measures” [1,7]. Much of the work that has been done provide commentaries on the state of immigrant children health care or explores issues of dental care among immigrant children in Western Canada [41,42,43,44,45], with limited attention to access to primary healthcare, specialist care, or health promotion [31], particularly as it relates to complex and chronic diseases [46]. Given that variations in access to primary and specialist care vary across the country, with provision differentially experienced depending on where a child lives and the regional availability of health care services in the area [47], more attention to immigrant children and family experiences access health care across Canada are needed in order to “improve immigrant’s experiences with navigating services which are fundamental to their health and wellbeing” [1,7]. As Carrol et al., (46 page S63) underscores there “must be more effort to help children and their families access the best quality care and treatments for lifelong conditions.” As rates of CIDs continue to increase across Canada with particular impacts on immigrant children and families [18,31,46,47], the objective of this paper is to address these needs by examining CID health care use and barriers to care among first and second generations South Asians immigrants in Ontario Canada. 

## 2. Context

This research was conducted in Ontario’s Greater Toronto Area (GTA) which comprises four neighboring municipal areas (i.e., Halton, Peel, York, and Durham), and 25 urban, suburban, and rural municipalities [48,49]. As one of Canada’s most diverse areas, reflecting both its current and historical role as an important immigration destination [48,49,50], over half of the GTA population (6.4 million) identifies as an immigrant, compared to 29.1% at the provincial scale [50]. Approximately 1.9 million (1,924,635) people identify as South Asian in the GTA, representing both the largest visible minority population in the GTA (32.3%), and the largest South Asian population in Ontario (84.6%) [50,51].

The Canadian South Asian diaspora is documented to experience a range of health and wellness needs ranging from higher rates of chronic diseases, lower rates of screening for a number of diseases, and greater risk of premature onset, increased prevalence and increased mortality from chronic diseases compared to the general public [52,53,54,55]. At the same time, recent reports indicate that although South Asian immigrants experience higher rates of CIDs than the general population, this group has low knowledge and awareness of CID symptoms, risk factors, and conditions [56].

Notwithstanding these inequalities, immigrants in the region commonly lack access to hospitals and health facilities and are more likely to experience discrimination, stigma, and racialization from staff compared to the general population [57,58]. This situation exists, despite the GTA housing the largest number of hospitals, general practitioners, and medical specialists in Ontario [51]. It is within this context that we examine the experiences of first and second generation South Asian immigrant children as they attempt to access health care to manage their CID.

## 3. Methods 

This paper draws from a larger qualitative research project comprising affected and unaffected first- and second-generation South Asians in the Greater Toronto Area (GTA). Affected individuals are those who personally manage a CID and/or have a child managing a CID (defined as a group of medical conditions characterized by chronic inflammation and varying levels of disability where one’s own immune system attacks and damages different parts of your body, requiring life-long treatment to control the impact on one’s life) whereas unaffected individuals are those who do not personally manage a CID and do not have a family member who manages a CID see [59,60,61]. 

To acquire deeper knowledge on the experiences navigating CID health care, affected populations are the focus of this paper. Two groups of individuals with medical history of CIDs were selected to participate in the research: (i) affected parents, defined as individuals who were born in a South Asian country and had a Canadian-born child diagnosed with a CID; and (ii) affected adult children (18 years and older) defined as Canadian born children diagnosed with a CID who had a South Asian mother born in a South Asian country. This project was part of a larger study that aimed to understand why Canadian born South Asian children have a higher prevalence of developing a CID than those who are born in South Asian. Thus, we focused on exploring the realities of affected children born in Canada. It was important to involve parents born in South Asia in order to explore their perceptions and experiences surrounding environmental changes imposed by global migration and chronic inflammatory diseases, along with their experiences navigating the Canadian health care system for their children [18]. Interview participants were not related. 

Purposeful sampling was used to acquire information- rich participants who could speak to the realities of managing a CID [62]. Snowball sampling was also used to identify additional participants [63]. Participants were recruited through partner organizations which included newcomer centers in the GTA and neighbourhood community organizations. We also advertised posters at higher education facilities in the GTA including the University of Toronto campuses and distributed recruitment posters to South Asian faith based and community organizations. Similar recruitment strategies may be beneficial in large-scale epidemiological studies, however, given our use of purposeful sampling, sampling strategies may need to change. 

A total of 24 semi structured in-depth interviews (i.e., 14 children, 10 parents) were conducted in English. Questions probed key themes related to their background, experiences receiving and coping with a CID diagnosis, access to medical care and services, awareness of sources of CID information, and perceptions of CID risk factors. For instance, participants were asked if they had a regular family doctor or specialized care, their challenges and barriers accessing medical care and related services, as well as financial or social constraints to receiving care. While children spoke to their own navigating the health system with a CID, parents answered questions based on their experiences using the health care system for their diagnosed child. Prior to conducting the interview, an interview location convenient to the participant was chosen. Before starting the interview, the researcher went through the consent form with the participant, reminding the individual of the research focus and their right to withdrawal at any time. The researcher also stressed that the interview was more conversational based, and the participant could skip any questions they chose not to answer. 

The semi-structured in-depth interviews lasted between 45 min to one hour. Of the affected parents, 12.5% identified as men and 87.5% identified as women and all lived in Canada for an average of 20–24 years. (See Table 1 for participant characteristics).

Prior to conducting this research, ethical approval was granted through the Human Subjects office at the University of Toronto (#32565). Thematic coding, analysis, and categorization of the interviews were conducted using NVIVO 11 software. The coding occurred in three sequential stages. First, open coding was used to distinguish the distinct concept and themes from the interview transcripts for further categorization. This was followed by axial coding, which was conducted to refine and categorize themes as a way to develop unique and central thematic categories from the data. Selective coding was then used to refine the data and develop unique and focal thematic categories for analysis [64,65,66]. Key themes were identified according to their (i) relevance to the research objectives; (ii) the predominance of the same theme across different types of participants; and (iii) frequency of mention [64]. 

This iterative process of classifying themes, developing linkages, and explanations led to saturation, whereby the themes were well described through the data [66]. To enhance consistency and reliability in the thematic analysis, the authors discussed the results, and consulted other members of the research team to ensure consensus in interpretation. The remaining sections discuss the experiences accessing CID health care among children and their parents. The key themes of access to a regular health care provider, and barriers to access health care including health system barriers; geographic barriers; competing needs; and knowledge, language and cultural barriers are used to present the results. Each quotation is identified with a participant type (i.e., child or parent), participant number and the CID that the child or parent is managing.

## 4. Results

### 4.1. Access to a Regular Health Care Provider 

When participants were asked about who they regularly sought health care from to manage their CID, participants highlighted a variety of care providers ranging from general practitioners (GP), specialists (i.e., endocrinologist, allergy specialist, neurologist, vasculitis specialist, breathing specialist, gastroenterologist), and alternative health care providers (i.e., homeopathic, home remedies, herbal, natural/organic) (see Table 2; note participants could provide multiple responses). Of the 24 participants, the majority of children (*n* = 11) and parents (*n* = 7) regularly sought health care from a GP, while very few children and parents reported no regular access to a GP (*n* = 3; *n* = 2 respectively). While most of the children (*n* = 8) indicated not regularly seeking health care from a specialist, half (*n* = 5) of the parents indicated regularly seeking care from a specialist for their children. 

While the majority of participants indicated that they desired to receive care from a specialist, due to long wait lists, administrative challenges (e.g., paperwork, phone calls, etc.), and a shortage of specialized care providers, they were unable to receive specialist care. In fact, all participants indicated they were more likely to regularly seek care from a GP since GPs were more common than specialists. As highlighted below, despite being told by their GP that specialized care was needed, multifaceted health system challenges meant participants commonly sought care from a GP:

*“She (daughter) can access the family doctor the fastest. It takes a long time but it’s faster than the specialists. Because the specialist has a really really long wait list. The GP said to see the specialist, but you can’t because the time and she needs care. Then what?”–Parent, 1073, type 1 diabetes*

Interestingly, just less than half of the children (*n* = 6) and parents (*n* = 4) indicated regularly seeking health care from an alternative provider. When asked why participants regularly sought support through alternative health care, most children and parents (*n* = 5, *n* = 3 respectively) indicated seeking care from these providers because they were more welcoming and receptive to patient needs than a formal health setting. Participants explained that since they had been dismissed and neglected when seeking CID care in a formalized setting, they were more inclined to seek care from an alternative provider. As the following participant explains, experiences of disrespect and carelessness towards patient needs left participants feeling neglected and yearning for supportive providers:

*“Ya she [daughter]) was complaining to him [GP]) all, most of the time that she had heart burn and those kinds of things, but he said its normal. He didn’t treat her right away. He never talks to us about this, the rheumatic problem, he never talk about this or tell us the problems. You don’t feel supported. This is why we use it (alternate medicine). At least they listen to your problems.”–Parent, 1026, rheumatoid arthritis*

### 4.2. General Barriers to Access Health Care for CID Management 

To unpack the experiences navigating formalized health care, we focused on participants’ experiences accessing a GP or specialist in the region. When participants were asked about the challenges that they experienced accessing their regular CID health care provider, participants identified a number of barriers including those centered around health system issues, geographic barriers, competing needs, and knowledge, language, and cultural barriers (see Table 3). 

#### 4.2.1. Health System Barriers

The most common barrier to access health care among children and parents relates to health system issues ranging from long wait times, few health care clinics, and the affordability of medicine. When discussing challenges accessing their provider, many children (*n* = 8) indicated that due to a lack of accessible GPs in their area, they would often seek care in walk in clinics even when they had a GP. Participants explained that once at a clinic, they were left to wait several hours before they could even see an intake nurse to take their personal medical information. This resulted in situations where some patients would attempt to wait for care, but due to pain and discomfort, would decide to leave without receiving help. As the following participant highlights, overbooked health care systems, a paucity of available clinics, and an overburdened health system create a situation where patients are unable to effectively manage their CID:

*“There are barriers in terms of accessibility and location, like just clinic wise. If the doctor isn’t available cause of overbooking, you have to find a clinic. And there aren’t as many clinics available, and then the wait times would be very long, you’re in pain so you could end up just leaving. You’re not helped, and it makes the problem worse. If it was a walk-in clinic and you didn’t get there at a certain period in the morning, then you could end up waiting like 4 hours if there was a bunch of people ahead of you. It’s a problem.”–Adult child, 1075, IBD*

Parents indicated similar concerns, highlighting how limited available health care exacerbated challenges related to CID management. Several parents discussed situations where their child needed immediate medical attention to manage their CID, but due to unavailable and overbooked physicians, they were unable to receive care from their provider. This meant that even when parents had a GP, some parents (*n* = 5) were left to navigate the complexity of CID complications, with little knowledge or expertise, all of which exacerbated stress and anxiety and further complicated CID management:

*“Yeah there was so many situations not only asthma but uh, where I thought my son needed attention for his breathing but we couldn’t receive it. The doctor wasn’t available, due to overbooking they said. So, you’re forced to manage on our own. It adds stress and makes the problem worse.”–parent, 1076, asthma*

Participants equally highlighted how a lack of available specialists in the GTA undermined their ability to effectively manage their CID. Several children (*n* = 7) indicated that although they and their parents would actively look for a specialist to help manage their CID and general health needs, they were unable to secure a provider. Although participants recognized the importance of regularly seeing a specialist to oversee their CID, renew prescriptions, and request tests to track and monitor the progression of their disease, several children highlighted how they did not have a regularly provider. As highlighted in the following excerpt, a lack of health care providers not only undermines the ability of a child to effectively manage their CID but further puts them at a higher risk of complications and even death:

*“I couldn’t find a family doctor for the longest time. The only reason I found the one I’m at is cause I was driving by and they had on their billboard accepting new patients. It was a huge barrier before that. I have a very severe nut allergy, so I need to carry an epi pen. I needed to renew my EpiPen, but I didn’t have a doctor to do it. I could have gone to any clinic, but I wanted a family doctor. They would know my history and problems. So I went without an active prescription for years. If I had an anaphylactic episode, it could have been bad.”–Adult child, 1035, type 1 diabetes*

The cost of medication was a further barrier that prohibited effective CID management for both children and parents. Participants often indicated how limited health insurance coverage, together with the costs of medication made CID management extremely difficult. While some children (*n* = 8) had health insurance from their parents or school, others had no coverage (*n* = 6), meaning that both children and their parents experienced financial struggles in order to obtain medication for their needs. As a result, children and their parents were left with additional stress and financial worries over their ability to maintain health over the long term. As the participant below underscores, although parents may try alleviating worries among children, financial stress can create unintended consequences for the psychosocial health of a child who is managing a CID:

*“I didn’t have health insurance. Well, my parents didn’t so I didn’t. I needed inhalers and they were really expensive. We all knew it was expensive, but my parents tried to downplay it. But I know it’s so expensive. But I needed it, so that’s it. It made me feel so bad, we were already struggling but it made it worse.”-Adult child, 1040, asthma*

Parents shared this sentiment, emphasizing how the cost of medication prohibited them from effectively caring for and managing their child’s CID. Several parents (*n* = 6) indicated that although they had some form of health insurance that could cover their children, the health insurance did not cover all of the medication, procedures and tests, meaning that they were forced to pay out of pocket if they were to manage their child’s care needs. Although many of the parents (*n* = 8) indicated having little available money to freely spend, they rationalized that their children’s immediate needs were more important than future obligations. Yet, as the participant below explains, since a CID is a lifelong condition, the costs associated with CID management can inflict challenges that last across the lifetime:

*“We have insurance but it doesn’t cover everything. We need to pay for the medication. It’s a dollar a pill. They need two pills a day. So that’s around 60 dollars extra a month. It’s just less than a thousand dollars a year. It may not be a lot when you look daily but it adds up. We have little free money to spend, but it’s necessary for my child. But this cost is never ending. So then what?”–Parent, 1073, type 1 diabetes*

As highlighted above, health system barriers can create a range of challenges for participants that not only make CID management worse but can create new complications that can undermine health and wellbeing of the child and their family system.

#### 4.2.2. Geographic Barriers

The second most common barrier to healthcare among both children and parents relates to challenges of distance to a provider, available transportation, proximity, traffic and commuting. Children frequently mentioned that due to limited personal vehicles and distance to health providers, they would take public transportation, such a bus or subway, to receive medical assistance. Yet, in situations when they were sick or in pain, participants explained being unable to use public transportation due to discomfort and the additional stress. While in some instances children indicated being able to find someone to drive them to a GP, in many situations they had no available means of reaching a provider, heightening their challenges and undermining their CID care:

*“Like there were times where I needed to go to the doctor because I was really sick, but I didn’t have any transportation to go there, other than taking the bus. I wasn’t willing to take the bus because I was too sick. It’s too hard and stressful. So I don’t go, but it creates more challenges cause you’re sick for days and other issues come up.”–Adult child, 1040, asthma*

Parents raised similar concerns impeding their access to health care. While some parents owned a vehicle, several (*n* = 6) highlighted having no access to a personal vehicle. As a result, they explained they needed to either take public transportation or find someone to transport both themselves and their sick child to care. Yet, due to pre-existing work obligations, costs of hospital parking and gasoline, parents often struggled to secure transportation to access care. As underlined in the following quotation, distance to a health care provider, along with additional opportunity costs and health care fees meant that parents would often resort to using public transportation, despite the pain this may inflict for their child:

*“It’s hard to find someone who can drive, especially during the day cause of work hours. If you’re lucky to find someone who will drive you there, then there’s costs of parking and somebody has to wait there. Parking is so much (expensive) and you feel bad cause the person is taking their time off. So we often resort to subway. I know doing that is hard on my child, especially when they’re so sick, but it’s that, or nothing.”–Parent, 1026, rheumatoid arthritis*

Other participants equally stressed how the lack of proximity of a health care provider, along with traffic and long commute distances limited their ability to effectively manage their CID. Some children (*n* = 5) explained that although they had a specialized care provider that would oversee their CID management, issues of geographic spawl, financial insecurity, and few specialized care providers, created a situation where children would decide not to seek care, even if they knew they needed it. As indicated in the subsequent excerpt, geographic inequities in the region, combined with a paucity of transportation networks and available care providers created a cycle of health mismanagement, poor health outcomes and uncertain futures:

*“It’s distance right. That’s the problem. Like, the neurologist is in Toronto, so it’s hard for me to get there. Especially if I don’t have a ride. I’m far from a transit line. And if you pay for a taxi, the fees add up. If there’s traffic, the toll keeps rolling and costs increase. So the distance and the costs, it’s a barrier. You can’t properly manage it (CID)and your left with more questions about your health. So it’s hard.”–Adult child, 1072, rheumatoid arthritis*

Parents similarly highlighted these barriers, noting how (un)available transportation, distance, and economic issues intersected to create a context of ambiguous care. Despite yearning to effectively manage their child’s illness, several parents (*n*= 7) stressed the layered challenges they encountered seeking care from a GP or specialist and the ways they impacted their child’s heath. As underscored in the following passage, intersecting economic, geographic, and medical inequities create further challenges for CID management:

*“There are so many issues. Travelling. Transportation. Financial. And the distance, it’s too far. We’re supposed to see the specialist once a month, but the location is a barrier. It’s not close and we have no available car, so we have to use a taxi. I don’t have a job it’s difficult to pay for transportation. Then if we need medication, you have to pay for transport to the pharmacy and back. So it all adds up and makes managing health very hard.”–Parent, 1073, type 1 diabetes*

As this section illustrates, even if children and parents can theoretically access health care, a range of intersecting challenges can make care unviable.

#### 4.2.3. Competing Needs

The third most frequently discussed barrier to care relates to competing needs. While the needs varied among children and parents, both groups indicated having to weigh personal needs against accessing CID health care. When discussing their experiences managing their CID, children highlighted how they needed to attend multiple appointments with doctors and specialists which impacted their attendance in school and ability to keep up with class work. While children recognized the need to attend weekly or monthly appointments with their health provider, they also recognized how attending frequent health appointments was negatively impacting their learning in school. As explained in the following quote, although children understood the need to seek health care to manage both their health and their parents stress, their desire to stay on track of their learning in school, created a barrier for some (*n* = 6) to access health care:

*“It was a difficult process, because now I’m like skipping school and am falling behind. My family is really stressed out with my health. That’s all they talk about. I’m meeting people that I’ve never met before, like dieticians, and every three months, going hospitals, doing more blood checkups and stuff. It’s a lot. So, there are times I didn’t go so I could go to school.”–Adult child, 1038, type 1 diabetes*

In contrast, parents often discussed issues related to work and personal household finances. Several parents raised challenges related to their need to attend work but also their need to take their child to requisite medical appointments. When discussing these issues, participants highlighted how they were often forced to pit their child’s health against their family’s financial health. While participants often prioritized their child’s health, several stressed how barriers tied to precarious employment landscapes, together with limited social support networks, created a situation of tension, anxiety, and ambiguous familial stability:

*“I can’t take time off in my job. It’s not possible or I may lose it. But the appointments are during the day, so it’s difficult. I have no one else to take him so I don’t know what to do. My child’s health or our financial health? My child is first, but I don’t know….”–Parent, 1032, rheumatoid arthritis*

Other parents equally emphasized the importance of employment in their access to health care. Several explained that since they were living on a tight budget, they had limited money to pay for transportation to health care, which in some cases was further exacerbated by lost family income due to their child’s CID. As a result, some parents were left to personally negotiate between attending their job to acquire sufficient money for transportation and livelihood needs but neglecting their child’s health or seeking health care but losing both financial security and opportunity costs. On the one hand, participants recognized the need to seek health care for their child, but on the other hand, they ultimately recognized that to access health care, they needed the money from employment. These situations not only inflicted more stress and hardship for parents and their children, but could create new challenges for CID management:

*“The appointments are during the day, I have to work. We need the money to pay the bills. She used to earn money too but she can’t earn now cause of the disease. So it’s tough. I know we need to go to the medical appointments, but I need to work so we have the money to actually go to the appointments and pay for everything else to manage the disease. Sometimes I have to work and we postpone care.”–Parent, 1027, IBD*

Tenuous financial situations were also highlighted as a barrier to manage health care. Several parents (*n* = 7) indicated that due to the instability of their jobs and limited savings, they had to make tough choices between purchasing medication to manage their child’s CID and going further in debt, or not acquiring medication, but watching their child suffer. Although participants acknowledged the potential financial difficulties that would follow, parents would often purchase the medication for their child, despite the potential of creating additional long term household consequences:

*“There was a pill he used when he was very small because he was getting asthma constantly. The pill itself was a dollar. I forget the name of it, it’s a really good pill, it helps him. But I had, you know a financial situation where I had to decide if it’s necessary to get those pills. We didn’t have available money and out jobs were not stable. But then I look at the way my son is suffering, so I say it’s okay to get those pills right. So I buy them, but it’s really expensive. So, we have to cut back a lot at home.”–Parent, 1076, asthma*

Competing educational, financial, and employment demands meant that individuals were left with difficult decisions–each decision generating new and uncertain challenges for the health and wellbeing of the child and family.

#### 4.2.4. Knowledge, Language, and Cultural Barriers

The fourth barrier characterizing participants’ access to healthcare relates to knowledge, language, and cultural barriers. When discussing their CID experience, several children highlighted how their limited knowledge about CIDs and/or its impact on the South Asian community hindered their knowledge of what was happening to their body, and as a result, made them think their CID challenges were normal. This meant that even if they were experiencing CID like symptoms, they were less inclined to seek health care since they thought their experiences were normal. Yet, as the participant explains in the following statement, once they acquired the knowledge of CIDs and what was occurring in their body, they could seek the right kind of information and were more motivated to pursue health care:

*“Before I didn’t have internet and didn’t have information. I didn’t know what was happening with my body so it was a barrier because I didn’t know what to do, or if I should go to health care. But certainly, with the help of the internet and just more access to information than before, like medical journals online, you have this huge wealth of information to look to, and that helps you don’t feel as in the dark. If it says I should see a doctor, then I do.”–Adult child, 1065, IBD*

Parents raised similar concerns surrounding how their limited knowledge of CIDs restricted their use of health care. While some parents (*n* = 5) indicated initially accessing health care to seek a preliminary health assessment for their child, they explained how their limited knowledge of CIDs, together with indifferent physicians created a situation where parents were not only left with limited insight into how to properly care for their child’s health, but also less motivated to seek further health care. As highlighted in the following excerpt, the perceived neglect of health workers made parents feel dismissed and less inclined to seek future health care:

*“In the beginning I didn’t have knowledge at all so I had a really hard time, I don’t know how to give him [child] the puffer, so most of the time he took it and it seems like he doesn’t know how to inhale it. I didn’t have the knowledge. And when we went to the doctor, they [doctors] didn’t explain so it was a barrier for sure. They hardly helped so I don’t want to deal with them next time.”–Parent, 1076, asthma*

Even still, children highlighted how their limited linguistic repertoire surrounding CID symptoms, not only left them unable to describe their health problems but also stifled their knowledge of appropriate management strategies. Throughout discussions, several children (*n* = 11) indicated that even when they could acquire some CID information, they faced several challenges related to informational interpretation. While some children (*n* = 4) indicated being unable to effectively use the information because the English terms were not translated, other children (*n* = 6) indicated being unable to use the information because the material was not explained in a straightforward manner they could understand. As a result, children explained feeling lost navigating health information as they contemplated if they should seek care or not:

*“The barriers depend on you and how much you know initially. You have to try to narrow things down, literally by the medical term, or side effect or symptom to get more information. But because we don’t have the language for it, we don’t know and the information there, it’s not translated. So we don’t know if we’re supposed to go to the hospital or if it’s nothing. So it’s a challenge.”–Adult child, 1065, rheumatoid arthritis*

Parents similarly stressed how linguistic issues impeding their use of health care. Although many parents had lived in Canada for many years, several (*n* = 6) indicated how their limited English language proficiency made health care access particularly challenging, especially when health workers would not take the time to explain issues. Although parents indicated how they would try to ask questions of health care providers to understand CID issues, they indicated that their concerns were often left unaddressed. This created a situation where parents were not only left feeling uncertain over the proper management of their child, but also apprehensive about seeking further health care:

*“Her (child) language is ok because she’s born here, she studied here, so she can communicate properly. But these are the things hard for me. I don’t have language for everything so it’s a challenge when we go to health care. They don’t even try to explain to me. So I’m not comfortable coming back all the time.”–Parent, 1058, IBD*

Issues of cultural continuity of care were also mentioned as a barrier to subsequent health care use. Several parents indicated that although they appreciated the health care received in a formal setting, they indicated that once in the community and attempting to manage their child’s CID, they faced several challenges such as limited culturally tailored community support. While some parents sought support through community groups at the direction of their physician, they indicated how these groups catered to specific populations, providing support in very culturally homogenous ways. This meant that even when participants attempted to access support services, culturally insensitive care made them feel isolated and less motivated to seek follow up support to assist their child. As highlighted below, even when parents desired to learn effective management techniques, culturally inappropriate support diminished parents’ willingness to seek out additional community support in the future:

*“I don’t know of any programs or support groups that apply to me. They don’t have specific information for South Asians. The information is too general. I want specific advice that applies to us, but they don’t have that. It doesn’t apply so we don’t know what to do to manage the disease. You just stop going.”–Parent, 1073, type 1 diabetes*

As this section illustrates, accessing health care is a challenging process, marked by inequities in knowledge, language, and cultural care, that can not only undercut effective CID management, but can also generate outcomes of poor health seeking behaviour.

## 5. Discussion

This study demonstrates that although CIDs disproportionately contributes to high rates of morbidity and disease complications among South Asian immigrant populations in Canada [12,28,67], first- and second-generation immigrant children and parents in this study face health system, geographic, interpersonal and knowledge barriers to access requisite care. While all participants recognized the importance of seeking regular care from a GP or a specialist to manage CID complications, most participants were unable to seek timely care when needed. Although participants expressed a desire to use specialized care, less than half of the participants could access a specialist on a regular basis. Yet even though over half of the participants had a regular GP, systemic, geographic and interpersonal barriers made supporting CID management extremely difficult, especially for those with limited financial security and/or family support.

The findings from this work suggest that while the GTA has the highest number of hospitals, general practitioners, and medical specialists in Ontario [68,69,70], health system barriers, geographic inequities, and competing needs mean South Asian immigrants are forced to delay care, seek care from alternative providers, or go without medical treatment, even in instances when they have a GP. Although participants had different lived realities and health care experiences, all participants stressed how barriers to health care made them feel more uncertain about their CID management and potential disease complications. The finding that parents had to make complex decisions over seeking health care for their child or go to work in order to raise sufficient funds for transport and basic familial necessities further underscores how systemic inequities among immigrants in the GTA [70,71,72,73] have created a situation where immigrants are unable to attain health equity.

According to current research, racialized immigrants in Toronto continue to be overrepresented in low-income jobs, with members of racialized groups representing 62% of all persons living in poverty [74]. Moreover, individuals who are racialized, unemployed, underemployed, or precariously employed in the region face financial challenges associated with paying for prescription medications since outpatient prescriptions are not covered by public funding [75,76]. This was the case in this study, where, due to limited financial security, participants did not have the access or ability to equip themselves with the medication and support needed to manage their CID. As Mahabir [77], (pg. 360) argues health care planning “must incorporate a broader system thinking that includes acknowledging systemic racism in the labor market and the resulting, devastating impacts of poverty on social conditions for racialized groups” in order to “bring into view the existence of an unequal, integrated systems of policies and laws that result in racial/ethnic stratification or structural racism.”

Prior studies have found that although barriers to access health care exist in the GTA and Ontario broadly, when health promotion interventions have been implemented among the general population in the region, they raise awareness and knowledge of health prevention, diseases, and disorders; increase patient use and uptake of health care services; and improve diagnostic pathways [78,79,80]. The results from this study however indicate that such interventions are not translating into improved health outcomes for first and second generation South Asian immigrants managing a CID. As the findings underscore, particular health care barriers unique to racialized immigrant populations, such as culturally insensitive health care support services, financial insecurity, limited social networks to assist with care, and/or medical racism, are not addressed nor considered when applying interventions to support the general population [81,82], indicating that participants in this study both lacked knowledge of CIDs and the capacity to utilize health services. A recent study on South Asian knowledge, attitudes, and perceptions of CIDs in the GTA found South Asian children and parents have low knowledge and awareness of CID symptoms, risk factors, and conditions [56]. This study builds on these findings to demonstrate how a lack of CID knowledge not only directly impacts one’s ability to access care and effectively manage a CID, but also how limited knowledge intersects with geographic, systemic, and personal inequities to create a situation of poor health outcomes.

Equally, the findings that participants chose alternate medicine over specialized care due to more receptive, supportive, and welcoming providers is indicative of the culturally insensitive care, racial discrimination, and overburdened health systems in Ontario, that have only been made worse during the pandemic [83,84,85,86]. These results are consistent with Mahabir’s [86] study of health care policies and practices on racial/ethnic groups in the GTA which found disrespect, mistreatment, negligent communication, and professional misconduct are main drivers shaping unequal access to health and health services among racial/ethnic minorities in the region, compounded during the pandemic. Yet, as the Ontario Government considers deregulating the College of Traditional Chinese Medicine Practitioners and Acupuncturists in Ontario with no consultation or discussion (see: [87,88]), it raises concerns that South Asian immigrants and others in need of care, and/or unable or unwilling to seek care from a GP or specialist may turn to unregulated health care or not seek care at all [89,90]. Thus, careful examination of how various experiences, sources and contexts of discrimination create and/or perpetuate inequities in access and quality of health care for immigrant minorities in Ontario is needed [58,91].

While this study brings attention to the barriers accessing CID health care among first and second South Asian immigrants, there are several study limitations. First, since the study was conducted with individuals living in the GTA, the experiences of health care use and barriers to care are context specific. That said, the findings point to issues applicable beyond the geography from which it emerges; particularly the impacts of socio-economic and structural inequities on health management, competing needs surrounding health care, and overburdened health systems to support CID management. Second, although this study was conducted with South Asia immigrants, it is not representative of the diversity of this population, with respect to ethnicity, religion, geography, and history. While multiple recruitment strategies were used, our sample does not reflect the intersectional experiences of South Asian individuals managing a CID. Lastly, the affected adult children and affected parents in this study were individuals who identified as women. Since our sample is gender imbalanced, we are unable to assess health care use and barriers to care differences between adult children, or mothers and fathers. Prior literature suggests that individuals who identify as women are more willing to discuss their health issues, participate in health research and act as health advocates for themselves and their families [92,93,94]. Thus, consideration to different recruitment strategies in more male dominated spaces could address some of these issues.

Future work that considers how barriers to care vary along intersectional axes of sex, gendered practices, and immigration status is needed to unpack the underlying mechanisms of privilege and disadvantage to provide evidence capable of designing public health policies that effectively reduce health inequities [95,96]. Equally, attention to the everyday forms of discrimination that impact realities and access to care are needed to enhance cultural competence and non-discriminatory care, improve linguistically accessible services and foster patient provider communication [91,97]. As knowledge continues to emerge on the uneven access and use of health services among immigrants in the GTA, Ontario, and in Canada [84,85,86], the results emphasize the need for a comprehensive health approach to address individual and system barriers to health care in order promote equitable client-centered care [11]. For instance, policies and programs that address socio-economic inequities, racial discrimination, and health system challenges in conjunction with programs that target health knowledge, personal needs and support services at the community scale through organization like the Multicultural Inter Agency Group of Peel, the Newcomer Center of Peel, and Toronto Public Health would not only enhance knowledge and awareness of CIDS but also address structural inequities to improve population health.

As the paper highlights, simply having a universal health care system in Canada does not translate into increased health care uptake without addressing broader social, economic, neighbourhood, built environment and systemic issues underpinning health realities. As Etowa [2,32] argues, “despite the emphasis on health equity of the Health Canada Act, research points to a disproportionate burden of difficulties accessing health care services among vulnerable populations in Canada.” Thus, more attention to the various societal conditions and racial practices that contribute to immigrant health disparities and predispose these populations to CIDs are needed to track and address race-based inequalities across health outcomes [98,99,100]. One way to improve immigrant health outcomes for example, is to tailor health care by assessing the patient’s social determinants of health while supporting medical care for racialized individuals and groups who self-define their priorities [101,102]. As the prevalence of CIDs continues to rise in Canada, with immigrants disproportionately impacted, attention to the multidimensional barriers to health care is urgently needed. Until that time, immigrants will continue to suffer the impacts of unequitable health care access.

## Figures and Tables

**Table 1:** Participant Characteristics.

	Number of Affected Adult Child (*n* = 14)	Percentage of Affected Parents (*n* = 10)
**Age (years)**		
18–24	12 (92.4%)	–
25–34	1 (7.7%)	–
35–44	–	2 (28.6%)
45–54	–	3 (42.9%)
55–64	–	2 (28.6%)
**Gender**		
Men	3 (23.1%)	1 (12.5%)
Women	10 (76.9%)	7 (87.5%)
**Country of Birth**		
Canada	14 (100%)	–
Sri Lanka	–	4 (44.4%)
India	–	5 (55.6%)
**Length of time residing in Canada**		
5–9 years	–	1 (12.5%)
10–14 years	–	1 (12.5%)
15–19 years	–	0
20–24 years	–	1 (12.5%)
25–29 years	–	4 (50%)
30–34 years	–	1 (12.5%)
**Type of CID**		
Type 1 diabetes	4 (29%)	3 (30%)
Inflammatory bowel disease (IBD)	3 (21%)	2 (20%)
Rheumatoid arthritis	3 (21%)	2 (20%)
Asthma	4 (29%)	3 (30%)

**Table 2:** Type of regular health care provider.

	General Practitioner (GP)	Specialist (Endocrinologist, Allergy Specialist, Neurologist, Vasculitis Specialist, Breathing Specialist, Dermatologist, Gastroenterologist)	Alternative Health Care (Homeopathic, Home Remedies, Herbal, Natural/Organic)
**Affected child**			
Yes	11	6	6
No	3	8	7
**Parent of affected child**			
Yes	7	5	4
No	2	3	5

**Table 3:** Barriers to access GPs and Specialists.

Participant Group	Barriers	# of Participants (# of Mentions)
**Affected Child**	**Yes, there are challenges and/or barriers**	14
	Health system barriers	12 (14)
	Geographic barriers	9 (13)
	Competing needs	6 (7)
	Knowledge, language and cultural barriers	3 (6)
**Parent of Affected Child**	**Yes, there are challenges and/or barriers**	10
	Health system barriers	8 (11)
	Geographic barriers	7 (11)
	Competing needs	6 (9)
	Knowledge, language and cultural barriers	5 (6)

## References

[1] Salami B., Olukotun M., Vastani M., Amodu O., Tetreault B., Obegu P.O., Plaquin J., Sanni O. (2022). Immigrant child health in Canada: A scoping review. BMJ Glob. Health.

[2] Markkula N., Cabieses B., Lehti V., Uphoff E., Astorga S., Stutzin F. (2018). Use of health services among international migrant children—A systematic review. Global Health.

[3] Volken T., Rüesch P. (2014). Health Status Inequality among Immigrants in Switzerland. Open J. Prev. Med..

[4] Klein J., Knesebeck O.V.D. (2018). Inequalities in health care utilization among migrants and non-migrants in Germany: A systematic review. Int. J. Equity Health.

[5] Statistics Canada (2003). Longitudinal Survey of Immigrants to Canada: Process, Progress and Prospects.

[6] Ahmad A. (2020). Ethnic discrimination against second-generation immigrants in hiring: Empirical evidence from a correspondence test. Eur. Soc..

[7] Kalich A., Heinemann L., Ghahari S. (2015). A Scoping Review of Immigrant Experience of Health Care Access Barriers in Canada. J. Immigr. Minor. Health.

[8] Gushulak B.D., Pottie K., Roberts J.H., Torres S., DesMeules M., on behalf of the Canadian Collaboration for Immigrant and Refugee Health Migration and health in Canada (2010). Health in the global village. Can. Med. Assoc. J..

[9] Zanchetta M.S., Poureslami I.M. (2006). Health literacy within the reality of immigrants’ culture and language. Can. J. Public Health.

[10] Koehn S., Neysmith S.M., Kobayashi K.M., Khamisa H. (2012). Revealing the shape of knowledge using an intersectionality lens: Results of a scoping review on the health and health care of ethnocultural minority older adults. Ageing Soc..

[11] Pandey M., Kamrul R., Michaels C.R., McCarron M. (2021). Identifying Barriers to Healthcare Access for New Immigrants: A Qualitative Study in Regina, Saskatchewan, Canada. J. Immigr. Minor. Health.

[12] Agrawal M., Burisch J., Colombel J.-F., Shah S.C. (2019). Viewpoint: Inflammatory Bowel Diseases Among Immigrants From Low- to High-Incidence Countries: Opportunities and Considerations. J. Crohn’s Colitis.

[13] Agrawal M., Shah S., Patel A., Pinotti R., Colombel J.-F., Burisch J. (2019). Changing epidemiology of immune-mediated inflammatory diseases in immigrants: A systematic review of population-based studies. J. Autoimmun..

[14] Slavich G.M. (2015). Understanding inflammation, its regulation, and relevance for health: A top scientific and public priority. Brain Behav. Immun..

[15] GBD 2017 Causes of Death Collaborators (2018). Global, regional, and national age-sex-specific mortality for 282 causes of death in 195 countries and territories, 1980–2017: A systematic analysis for the Global Burden of Disease Study 2017. Lancet.

[16] Bennett J.M., Reeves G., Billman G.E., Sturmberg J.P. (2018). Inflammation–nature’s way to efficiently respond to all types of challenges: Implications for understanding and managing “the epidemic” of chronic diseases. Front. Med..

[17] Agrawal M., Corn G., Shrestha S., Nielsen N.M., Frisch M., Colombel J.-F., Jess T. (2020). Inflammatory bowel diseases among first-generation and second-generation immigrants in Denmark: A population-based cohort study. Gut.

[18] Copeland J.K., Chao G., Vanderhout S., Acton E., Wang P.W., Benchimol E.I., El-Sohemy A., Croitoru K., Gommerman J.L., Guttman D.S. (2021). The Impact of Migration on the Gut Metagenome of South Asian Canadians. Gut Microbes.

[19] El-Gabalawy L.C., Guenther C.N. (2010). Bernstein, Epidemiology of immune-mediated inflammatory diseases: Incidence, prevalence, natural history, and comorbidities. J. Rheumatol. Suppl..

[20] Ng S.C., Shi H.Y., Hamidi N., Underwood F.E., Tang W., Benchimol E.I., Panaccione R., Ghosh S., Wu J.C.Y., Chan F.K.L. (2017). Worldwide incidence and prevalence of inflammatory bowel disease in the 21st century: A systematic review of population-based studies. Lancet.

[21] Torres J., Mehandru S., Colombel J.F., Peyrin-Biroulet L. (2017). Crohn’s disease. Lancet.

[22] Ungaro F., Rubbino F., Danese S., D’Alessio S. (2017). Actors and Factors in the Resolution of Intestinal Inflammation: Lipid Mediators As a New Approach to Therapy in Inflammatory Bowel Diseases. Front. Immunol..

[23] Placha D., Jampilek J. (2021). Chronic inflammatory diseases, anti-inflammatory agents and their delivery nanosystems. Pharmaceutics.

[24] Canadian Institutes of Health Research (2019). Inflammation in Chronic Disease–Featured Researchers. https://cihr-irsc.gc.ca/e/49032.html.

[25] I Benchimol E., Mack D.R., Guttmann A., Nguyen G.C., To T., Mojaverian N., Quach P., Manuel D.G. (2015). Inflammatory Bowel Disease in Immigrants to Canada and Their Children: A Population-Based Cohort Study. Am. J. Gastroenterol..

[26] Jones J.L., Nguyen G.C., I Benchimol E., Bernstein C.N., Bitton A., Kaplan G.G., Murthy S.K., Lee K., Cooke-Lauder J., Otley A.R. (2018). The Impact of Inflammatory Bowel Disease in Canada 2018: Quality of Life. J. Can. Assoc. Gastroenterol..

[27] Yousef S., Colman I., Papadimitropoulos M., Manuel D., Hossain A., Faris M., Wells G.A. (2022). Vitamin D and Chronic Diseases among First-Generation Immigrants: A Large-Scale Study Using Canadian Health Measures Survey (CHMS) Data. Nutrients.

[28] Kaplan G.G., Bernstein C.N., Coward S., Bitton A., Murthy S.K., Nguyen G.C., Lee K., Cooke-Lauder J., Benchimol E.I. (2019). The Impact of Inflammatory Bowel Disease in Canada 2018: Epidemiology. J. Can. Assoc. Gastroenterol..

[29] Benchimol E.I., Manuel D.G., Guttmann A., Nguyen G.C., Mojaverian N., Quach P., Mack D.R. (2014). Changing Age Demographics of Inflammatory Bowel Disease in Ontario, Canada: A population-based cohort study of epidemiology trends. Inflamm. Bowel Dis..

[30] Pinsk V., Lemberg D.A., Grewal K., Barker C.C., Schreiber R.A., Jacobson K. (2007). Inflammatory Bowel Disease in the South Asian Pediatric Population of British Columbia. Am. J. Gastroenterol..

[31] Salami B., Mason A., Salma J., Yohani S., Amin M., Okeke-Ihejirika P., Ladha T. (2020). Access to healthcare for immigrant children in Canada. Int. J. Environ. Res. Public Health.

[32] Etowa J., Hyman I., Dabone C., Mbagwu I., Ghose B., Sano Y., Osman M., Mohamoud H. (2021). Strengthening the Collection and Use of Disaggregated Data to Understand and Monitor the Risk and Burden of COVID-19 Among Racialized Populations. Can. Stud. Popul..

[33] Hyman I., Patychuk D., Zaidi Q., Kljujic D., Shakya Y., Rummens J., Creatore M., Vissandjee B. (2012). Self-management, health service use and information seeking for diabetes care among recent immigrants in Toronto. Chronic Dis. Inj. Can..

[34] Durbin A.P., Moineddin R., Lin E., Steele L.S., Glazier R.H. (2015). Mental health service use by recent immigrants from different world regions and by non-immigrants in Ontario, Canada: A cross-sectional study. BMC Health Serv. Res..

[35] Tu J.V., Chu A., Rezai M.R., Guo H., Maclagan L.C., Austin P.C., Booth G.L., Manuel D.G., Chiu M., Ko D.T. (2015). Incidence of Major Cardiovascular Events in Immigrants to Ontario, Canada. Circulation.

[36] Harrington D.W., Wilson K., Rosenberg M., Bell S. (2013). Access granted! barriers endure: Determinants of difficulties accessing specialist care when required in Ontario, Canada. BMC Health Serv. Res..

[37] (2011). Newcomer Health. https://www.toronto.ca/legdocs/mmis/2011/hl/bgrd/backgroundfile-42361.pdf.

[38] Glazier R.H., Tepper J., Agha M.M., Moineddin R. (2006). Primary care in disadvantaged populations. Primary Care in Ontario: ICES Atlas.

[39] Patil C.L., Maripuu T., Hadley C., Sellen D.W. (2012). Identifying Gaps in Health Research among Refugees Resettled in Canada. Int. Migr..

[40] Vang Z.M., Sigouin J., Flenon A., Gagnon A. (2017). Are immigrants healthier than native-born Canadians? A systematic review of the healthy immigrant efect in Canada. Ethn. Health..

[41] Amin M., Perez A. (2012). Is the wait-for-patient-to-come approach suitable for African newcomers to Alberta, Canada?. Community Dent. Oral Epidemiol..

[42] Fellin M., King G., Esses V., Lindsay S., Klassen A. (2013). Barriers and facilitators to health and social service access and utilization for immigrant parents raising a child with a physical disability. Int. J. Migr. Health Soc. Care.

[43] Hoover J., Vatanparast H., Uswak G. (2017). Risk Determinants of Dental Caries and Oral Hygiene Status in 3–15 Year-Old Recent Immigrant and Refugee Children in Saskatchewan, Canada: A Pilot Study. J. Immigr. Minor. Health.

[44] Werneck R., Lawrence H.P., Kulkarni G.V., Locker D. (2008). Early childhood caries and access to dental care among children of Portuguese-speaking immigrants in the city of Toronto. J. Can. Dent. Assoc..

[45] Barozzino T. (2010). Immigrant health and the children and youth of Canada: Are we doing enough?. Health Q..

[46] Carroll M.W., Kuenzig M.E., Mack D.R., Otley A.R., Griffiths A.M., Kaplan G.G., Bernstein C.N., Bitton A., Murthy S.K., Nguyen G.C. (2018). The Impact of Inflammatory Bowel Disease in Canada 2018: Children and Adolescents with IBD. J. Can. Assoc. Gastroenterol..

[47] El-Matary W., Benchimol E.I., Mack D., Huynh H.Q., Critch J., Otley A., Deslandres C., Jacobson K., Debruyn J., Carroll M.W. (2017). Allied Health Professional Support in Pediatric Inflammatory Bowel Disease: A Survey from the Canadian Children Inflammatory Bowel Disease Network—A Joint Partnership of CIHR and the CH.I.L.D. Foundation. Can. J. Gastroenterol. Hepatol..

[48] Statistics Canada (2021). Updated Content for the 2021 Census of Population: Immigration, Ethnocultural Diversity and Languages in Canada. https://www12.statcan.gc.ca/census-recensement/2021/ref/98-20-0001/982000012020002-eng.cfm.

[49] Statistics Canada (2021). Population Estimates, July 1, by Census Metropolitan Area and Census Agglomeration, 2016 Boundaries. https://www150.statcan.gc.ca/t1/tbl1/en/tv.action?pid=1710013501.

[50] Statistics Canada’s Population Estimates: Sub Provincial Areas, July 1, 2020. https://www150.statcan.gc.ca/n1/daily-quotidien/210114/dq210114a-eng.htm.

[51] Statistics Canada (2016). Census Profile, 2016 Census Toronto [Census Metropolitan Area], Ontario and Ontario. https://www12.statcan.gc.ca/census-recensement/2016/dp-pd/prof/details/page.cfm?Lang=E&Geo1=CMACA&Code1=535&Geo2=PR&Code2=35&Data=Count&SearchText=toronto&SearchType=Begins&SearchPR=01&B1=All&TABID=1.

[52] Tiwari S.K., Wang J. (2008). Ethnic differences in mental health service use among White, Chinese, South Asian and South East Asian populations living in Canada. Soc. Psychiatry.

[53] D’Silva C., Hafleen N., Mansfield E., Martel S., Fierheller D., Banerjee A., Malhotra G., Mutta B., Dhillon P., Hasan Z. (2022). Service Provider Perspectives on Exploring Social Determinants of Health Impacting Type 2 Diabetes Management for South Asian Adults in Peel Region, Canada. Can. J. Diabetes.

[54] Rana A., de Souza R.J., Kandasamy S., Lear S.A., Anand S.S. (2014). Cardiovascular risk among South Asians living in Canada: A systematic review and meta-analysis. CMAJ Open.

[55] Fernando E., Razak F., Lear S.A., Anand S.S. (2015). Cardiovascular Disease in South Asian Migrants. Can. J. Cardiol..

[56] Rishworth A.C., Niraula A., Cao T., Lay J.C., Ferrari J., Zaman S., Wilson K. (2022). Knowledge, risk perceptions and practices surrounding chronic inflammatory diseases among first and second generation South Asian immigrants parents and children. Int. J. Migr. Health Soc. Care.

[57] Islam T., Selvaratnam I., Shan N., Building an Effective South Asian Health Strategy in Ontario (2013). In 3rd Annual Health Equity Conference, Council of Agencies Serving South Asians (CASSA). https://pchs4u.com/documents/research-reports-andresources/CASSAs-South-Asian-Strategy-Report.Pdf.

[58] Pollock G., Newbold B., Lafrenière G., Edge S. (2011). Perceptions of Discrimination in Health Services Experienced by Immigrant Minorities in Ontario.

[59] Furman D., Campisi J., Verdin E., Carrera-Bastos P., Targ S., Franceschi C., Ferrucci L., Gilroy D.W., Fasano A., Miller G.W. (2019). Chronic inflammation in the etiology of disease across the life span. Nat. Med..

[60] Pahwa R., Goyal A., Jialal I. (2022). Chronic Inflammation. Treasure Island (FL): StatPearls Publishing. https://www.ncbi.nlm.nih.gov/books/NBK493173/.

[61] Regence. What is Chronic Inflammatory Disease? 2021. https://www.hca.wa.gov/assets/pebb/ump-clinical-pathways-chronic-inflammatory-disease.pdf.

[62] Palinkas L.A., Horwitz S.M., Green C.A., Wisdom J.P., Duan N., Hoagwood K. (2015). Purposeful Sampling for Qualitative Data Collection and Analysis in Mixed Method Implementation Research. Adm. Policy Ment. Health Ment. Health Serv. Res..

[63] Parker C., Scott S., Geddes A. (2019). Snowball Sampling.

[64] Huberman M., Miles M.B. (2002). The Qualitative Researcher’s Companion.

[65] Holton J.A. (2012). The Coding Process and Its Challenges. SAGE Handbook of Grounded Theory.

[66] Dey M., Yadav D.P., Mahata S.K., Mondal A., Sahana S. An Improved Approach of Cryptography using Triangulation and MSB Iteration Technique. Proceedings of the International Journal of Computer Applications, Special Issue of 1st International Conference on Computing, Communication and Sensor Networks.

[67] Park S.-W., Kim T.J., Lee J.Y., Kim E.R., Hong S.N., Chang D.K., Yang M., Kim S., Shin M.-H., Kim Y.-H. (2018). Comorbid immune-mediated diseases in inflammatory bowel disease: A nation-wide population-based study. Aliment. Pharmacol. Ther..

[68] Burisch J., Jess T., Egeberg A. (2019). Incidence of Immune-Mediated Inflammatory Diseases Among Patients With Inflammatory Bowel Diseases in Denmark. Clin. Gastroenterol. Hepatol..

[69] CMA (Canadian Medical Association) (2021). A struggling System Understanding the Health Care Impacts of the Pandemic. https://www.cma.ca/sites/default/files/pdf/health-advocacy/Deloitte-report-nov2021-EN.pdf.

[70] Public Health Agency of Canada (2018). Public Health Agency of Canada. Key Health Inequalities in Canada: A National Portrait. Pan-Canadian Health Inequalities Reporting Initiative. https://www.canada.ca/content/dam/phac-aspc/documents/services/publications/science-research/key-health-inequalities-canada-national-portraitexecutive-summary/hir-full-report-eng.pdf.

[71] Toronto Public Health (2013). Racialization and Health Inequities in Toronto. http://www.toronto.ca/legdocs/mmis/2013/hl/bgrd/backgroundfile-62904.pdf.

[72] Siddiqi A., Shahidi F.V., Ramraj C., Williams D.R. (2017). Associations between race, discrimination and risk for chronic disease in a population-based sample from Canada. Soc. Sci. Med..

[73] Veenstra G., Patterson A.C. (2016). South Asian-White health inequalities in Canada: Intersections with gender and immigrant status. Ethn. Health.

[74] Colour of Poverty (2019). Racialized Poverty in Income and Social Assistance. https://colourofpovertyca.files.wordpress.com/2019/03/cop-coc-fact-sheet-6-racialized-poverty-in-income-social-assistance-1.pdf.

[75] Chowdhury M.Z., Chowdhury M.A. (2018). Canadian health care system: Who should pay for all medically beneficial treatments? A burning issue. Int. J. Health Serv..

[76] Cheff R., Hill M., Iveniuk J. (2019). Who Benefits? Gaps in Medication Coverage for Ontario Workers. Wellesley Institute. https://www.wellesleyinstitute.com/wp-content/uploads/2019/12/Coverage-Gaps-for-Ontario-Workers_EMBARGO_27.11.19.pdf.

[77] Mahabir D.F., O’Campo P., Lofters A., Shankardass K., Salmon C., Muntaner C. (2021). Classism and Everyday Racism as Experienced by Racialized Health Care Users: A Concept Mapping Study. Int. J. Health Serv..

[78] Gladstone D.J., Rodan L.H., Sahlas D.J., Lee L., Murray B., Ween J.E., Perry J.R., Chenkin J., Morrison L.J., Beck S. (2009). A Citywide Prehospital Protocol Increases Access to Stroke Thrombolysis in Toronto. Stroke.

[79] Kamali M., Sivapalan S., Kata A., Kim N., Shanmugalingam N., Duku E., Zwaigenbaum L., Georgiades S. (2022). Program evaluation of a pilot mobile developmental outreach clinic for autism spectrum disorder in Ontario. BMC Health Serv. Res..

[80] Ballios B.G., Park T., Chaudhary V., Hurley B., Kosar S., Sheidow T., Cruess A., Brent M.H., Glazier R., Wong D.T. (2020). Identifying gaps in patient access to diabetic screening eye examinations in Ontario: A provincially representative cross-sectional study. Can. J. Ophthalmol..

[81] Diaz E., Ortiz-Barreda G., Ben-Shlomo Y., Holdsworth M., Salami B., Rammohan A., Chung R.Y.-N., Padmadas S.S., Krafft T. (2017). Interventions to improve immigrant health. A scoping review. Eur. J. Public Health.

[82] Handtke O., Schilgen B., Mösko M. (2019). Culturally competent healthcare—A scoping review of strategies implemented in healthcare organizations and a model of culturally competent healthcare provision. PLoS ONE.

[83] Shang Z., Kim J.Y., Cheng S.O. (2021). Discrimination experienced by Asian Canadian and Asian American health care workers during the COVID-19 pandemic: A qualitative study. CMAJ Open.

[84] El-Dassouki N., Wong D., Toews D.M., Gill J., Edwards B., Orchanian-Cheff A., Neves P., Marshall L.-J., Mucsi I. (2021). Barriers to Accessing Kidney Transplantation Among Populations Marginalized by Race and Ethnicity in Canada: A Scoping Review Part 2—East Asian, South Asian, and African, Caribbean, and Black Canadians. Can. J. Kidney Health Dis..

[85] Chen J.A., Zhang E., Liu C.H. (2020). Potential Impact of COVID-19–Related Racial Discrimination on the Health of Asian Americans. Am. J. Public Health.

[86] Mahabir D.F., O’Campo P., Lofters A., Shankardass K., Salmon C., Muntaner C. (2021). Experiences of everyday racism in Toronto’s health care system: A concept mapping study. Int. J. Equity Health.

[87] CBC (2022). Don’t Scrap College Regulating Traditional Chinese Medicine, Critics Warn Province. https://www.cbc.ca/news/canada/toronto/practitioners-push-back-deregulate-chinese-medicine-1.6371315.

[88] Global News (2022). Ontario Planning to Deregulate Traditional Chinese Medicine, Acupuncture. https://globalnews.ca/news/8654667/ontario-deregulate-traditional-chinese-medicine/.

[89] College of Nurses of Ontario (2013). Working With Unregulated Care Providers Updated. Practical Guideline. https://www.cno.org/globalassets/docs/prac/41014_workingucp.pdf.

[90] Afzal A., Stolee P., Heckman G.A., Boscart V.M., Sanyal C. (2018). The role of unregulated care providers in Canada-A scoping review. Int. J. Older People Nurs..

[91] Zghal A., El-Masri M., McMurphy S., Pfaff K. (2020). Exploring the Impact of Health Care Provider Cultural Competence on New Immigrant Health-Related Quality of Life: A Cross-Sectional Study of Canadian Newcomers. J. Transcult. Nurs..

[92] Glass D.C., Kelsall H.L., Slegers C., Forbes A., Loff B., Zion D., Fritschi L. (2015). A telephone survey of factors affecting willingness to participate in health research surveys. BMC Public Health.

[93] Levinson W., Kao A., Kuby A., Thisted R.A. (2005). Not all patients want to participate in decision making: A national study of public preferences. J. Gen. Intern. Med..

[94] Sherman D.W. (2019). A Review of the Complex Role of Family Caregivers as Health Team Members and Second-Order Patients. Healthcare.

[95] Wandschneider L., Miani C., Razum O. (2022). Decomposing intersectional inequalities in subjective physical and mental health by sex, gendered practices and immigration status in a representative panel study from Germany. BMC Public Health.

[96] Richter S., Vallianatos H., Green J., Obuekwe C. (2020). Intersection of Migration and Access to Health Care: Experiences and Perceptions of Female Economic Migrants in Canada. Int. J. Environ. Res. Public Health.

[97] Ramos-Roure F., Feijoo-Cid M., Manresa-Dominguez J.M., Segura-Bernal J., García-Sierra R., Fernández-Cano M.I., Toran-Monserrat P. (2021). Intercultural communication between long-stay immigrants and Catalan primary care nurses: A qualitative approach to rebalancing power. Int. J. Environ. Res. Public Health.

[98] Anyane-Yeboa A., Quezada S., Rubin D.T., Balzora S. (2022). The Impact of the Social Determinants of Health on Disparities in Inflammatory Bowel Disease. Clin. Gastroenterol. Hepatol..

[99] Datta G., Siddiqi A., Lofters A. (2021). Transforming race-based health research in Canada. Can. Med. Assoc. J..

[100] Lin S. (2021). Access to health care among racialised immigrants to Canada in later life: A theoretical and empirical synthesis. Ageing Soc..

[101] Corneau S., Stergiopoulos V. (2012). More than being against it: Anti-racism and anti-oppression in mental health services. Transcult. Psychiatry.

[102] Goel R., Buchman S., Meili R., Woollard R. (2016). Social accountability at the micro level: One patient at a time. Can. Fam. Physician.

